# Eliminating residual iPS cells for safety in clinical application

**DOI:** 10.1007/s13238-015-0170-4

**Published:** 2015-06-04

**Authors:** Shigeo Masuda, Shigeru Miyagawa, Satsuki Fukushima, Nagako Sougawa, Kaori Okimoto, Chika Tada, Atsuhiro Saito, Yoshiki Sawa

**Affiliations:** Department of Cardiovascular Surgery, Osaka University Graduate School of Medicine, Osaka, 565-0871 Japan

First-in-human clinical trial for age-related macular degeneration using iPSC-derived retinal pigment epithelium (RPE) cells was conducted in 2014, showing no serious adverse effects to date, including tumor formation. This appears to be attributable to relatively small number of transplanted cells, and distinct morphology of RPE cells; it might be relatively easy to minimize contamination of undifferentiated cells. However, in the case of clinical trial using iPSC-derived cardiomyocytes, 10^8^–10^9^ cells would be required for transplantation; strict control of residual undifferentiated cells should be necessary for safer clinical application.

**Figure 1:**
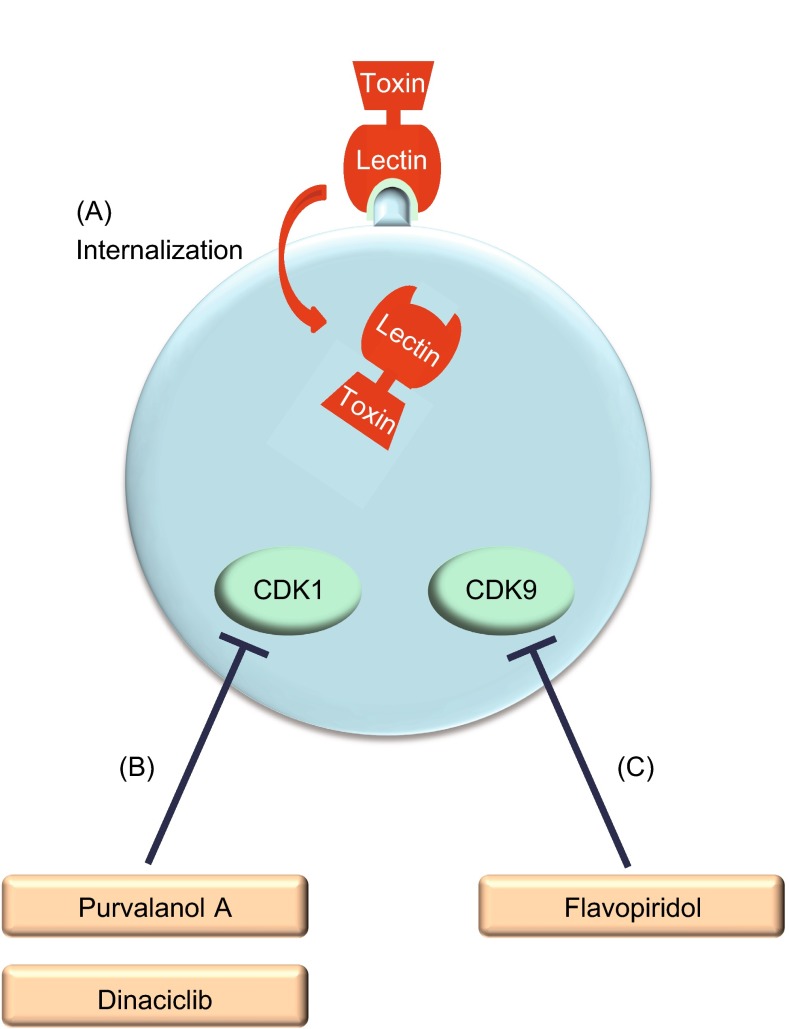
**Recent advances in selective elimination of human and/or mouse pluripotent stem cells**. (A) A recombinant lectin-toxin fusion protein (rBC2LCN-PE23) preferentially binds to and is internalized by human pluripotent stem cells, thus killing human pluripotent stem cells selectively (Tateno et al. 2015). (B) CDK1 inhibitors (Purvalanol A and Dinaciclib) are potent in inducing apoptosis in human and mouse ESCs. Treatment with a CDK1 inhibitor is demonstrated to prevent tetatoma formation (Huskey et al. 2015). (C) A CDK9 inhibitor (Flavopiridol) is shown to decrease Nanog and c-Myc expressions in mouse ESCs (Wu et al. 2015). It remains to be determined whether CDK9 inhibition results in prevention of teratoma formation

Accumulating evidence demonstrates several interesting methods of depleting undifferentiated cells, as shown previously (Masuda et al., 2014). We can classify these methods as positive selection or negative selection; in positive selection, differentiated cells that are positive for differentiation markers would be collected, whereas in negative selection, undifferentiated cells would be discarded, thus enabling to eliminate tumorigenic cells from iPSC-derived products. For negative selection, in most cases, molecular-targeted drugs have been used to kill iPSCs, such as small chemicals or antibodies (especially those conjugated with cytotoxic drugs). Very recently, three papers (Tateno et al., 2015; Huskey et al., 2015; Wu et al., 2015) have been published, demonstrating that novel different methods might be useful for removing tumorigenic iPSCs (Table 1) (Fig. 1).

**Table 1:** **Novel methods for putative prevention of teratoma formation**

	Tateno et al. (2015)	Huskey et al. (2015)	Wu et al. (2015)
Chemical or protein	Lectin-toxin fusion protein	Chemical	Chemical
Mode of action	Lectin-binding to iPSCs Cargo of cytotoxic agent	CDK1 inhibition	CDK9 inhibition
Drug	rBC2LCN-PE23	Purvalanol A Dinaciclib	Flavopiridol

Previously, Tateno and colleagues discovered that a lectin, called rBC2LCN, binds to human iPSCs and embryonic stem cells (ESCs), but not to differentiated cells (Tateno et al. 2011). Lectin is known to be protein that binds to glycan structure. Recently, they newly generated a recombinant lectin-toxin fusion protein (rBC2LCN-PE23) (Tateno et al. 2015); a catalytic domain of *Pseudomonas aeruginosa* exotoxin A was fused with rBC2LCN to eliminate human iPSCs. Notably, lectin-toxin fusion protein (rBC2LCN-PE23) could bind to and successfully be internalized by human iPSCs, suggesting that lectin (rBC2LCN) could be useful for delivery of cytotoxic agents into human iPSCs. Furthermore, within 24 h, complete elimination of human iPSCs was achieved by culture in the presence of lectin-toxin fusion protein, indicating that rBC2LCN-PE23 exerts a robust cytotoxic effect on human iPSCs. On the other hand, it has a negligible cytotoxic effect on differentiated cells, thereby enabling selective elimination of human iPSCs in a mixed cell population (Tateno et al. 2015).

In the present study by Tateno and colleagues, although selective elimination of human iPSCs was examined in a mixed cell population (where human fibroblasts were spiked by human iPSCs) (Tateno et al. 2015), differentiated cells (derived from human iPSCs) containing undifferentiated cells should be examined precisely in the future. For example, even among partially differentiated cells that lost lectin-binding (i.e. “lectin-negative” cells), there may be a risk of residual tumorigenic potential. Another issue to be addressed is that “bystander effect” of lectin-toxin fusion protein should be investigated; it would be checked whether cleaved toxin released from dying cells might affect surrounding cell’s viability. If we are able to replace exotoxin A with other toxins (proteins) for fusion, it would be interesting. Next issue is that, from the view point of 3D culture, it would be rather difficult for lectin-toxin fusion protein to be internalized into 3D tissues.

Second, Huskey and colleagues have identified that cyclin-dependent kinase (CDK) 1 (and its binding partners: cyclins A2 and B1/B2) would be critical for cell proliferation and survival in mouse and human ESCs by screening of siRNA knockdown (Huskey et al. 2015). The authors demonstrated that purvalanol A, a CDK1 inhibitor, could preferentially induce cell death in ESCs while sparing differentiated cells. In the present study, the authors used an alternative CDK inhibitor, dinaciclib, developed by Merck, with improved pharmacokinetic properties compared to purvalanol A. Dinaciclib inhibits CDK1, 2, 5, and 9, and is currently in clinical trials against multiple tumors. Although Ibrance^®^ by Pfizer (Palbociclib) (a CDK4/6 inhibitor) has been recently approved by FDA for the first time as a CDK inhibitor, CDK4/6 inhibition is known to be compensated by CDK1 activity. Anyway, there is unique dependency of ESCs on CDK1 activity (Huskey et al. 2015).

Third, Wu and colleagues investigated whether BRD4, a member of BET family, interacts with pluripotency-associated transcription factors (Wu et al. 2015). BRD4 is known to form active transcription complex, which consists of CDK9, CYCLIN T, and BRD4. Therefore, the authors treated mouse ESCs with flavopiridol, a CDK9 inhibitor, and observed that Nanog and c-Myc expressions were greatly decreased (Wu et al. 2015). Further elucidation will be needed to examine whether CDK9 inhibition is associated with ESC survival.

When we utilize iPSC-derived cells for clinical application, there are two types of tumor cells; a tumor (i.e. benign teratoma) derived from undifferentiated cells, and a tumor derived from differentiated cells. The latter may be malignant tumors, potentially with several mutations. Although we can prevent teratoma formation (the former part) by using emerging technology as mentioned above, it would be essential to pre-examine genome sequence of iPSC-derived products in order to prevent the occurrence of malignant tumors (the latter part). In summary, now, we can choose or combine several ways of preventing teratoma formation, depending on efficacy and safety/toxicity, via *ex vivo* purging of undifferentiated cells.
